# Development and Pilot Study of a Pediatric Screening for Feeding and Swallowing Disorders in Infants and Children: The Pediatric Screening–Priority Evaluation Dysphagia (PS–PED)

**DOI:** 10.3390/children10040638

**Published:** 2023-03-29

**Authors:** Antonella Cerchiari, Marco Tofani, Carolina Giordani, Silvia Franceschetti, Eleonora Capuano, Francesca Pizza, Gessica Della Bella, Massimiliano Raponi, Giorgia Biondo

**Affiliations:** 1 Management and Diagnostic Innovations & Clinical Pathways Research Area, Neurorehabilitation and Adapted Physical Activity Day Hospital, Bambino Gesù Children’s Hospital, 00165 Rome, Italy; carolina.giordani@opbg.net (C.G.); silvia.franceschetti@opbg.net (S.F.); eleonora.capuano@opbg.net (E.C.); francesca.pizza@opbg.net (F.P.); gessica.dellabella@opbg.net (G.D.B.); giorgia.biondo@opbg.net (G.B.); 2 Department of Human Neurosciences, Sapienza University of Rome, 00183 Rome, Italy; 3 Management and Diagnostic Innovations & Clinical Pathways Research Area, Professional Development, Continuous Education and Research Service, Bambino Gesù Children’s Hospital, IRCCS, 00165 Rome, Italy; 4 Management and Diagnostic Innovations & Clinical Pathways Research Area, Medical Directorate, Bambino Gesù Children’s Hospital, IRCCS, 00165 Rome, Italy; massimiliano.raponi@opbg.net

**Keywords:** Delphi, screening, assessment tool, dysphagia, feeding disorders, pediatrics

## Abstract

Feeding and swallowing disorders (FSD) are common during childhood, with a prevalence of 85% in children with neurodevelopmental disorders. A comprehensive screening is essential to identify FSD and improve health outcomes in a clinical setting. This study aims to develop a new Pediatric Screening tool capable of identifying FSD. This screening tool was developed in three steps: selecting variables based on clinical experience, searching the literature and finding agreement between experts with a two-round Delphi study. This process, which reached 97% of agreement between experts, led to the development of the Pediatric Screening–Priority Evaluation Dysphagia (PS–PED). PS–PED comprises 14 items divided into three main domains: clinical history, health status and feeding condition. We also carried out a pilot test for measuring internal consistency, as measured with Cronbach Coefficient alpha. Concurrent validity, as measured with Pearson correlation coefficient, was tested using a videofluoroscopy swallow study (VFSS) classified with the Penetration Aspiration Scale (PAS). The pilot test was conducted on 59 children with different health conditions. Our findings showed good internal consistency (alpha = 0.731), and a strong linear correlation with PAS (Pearson 0.824). Furthermore, comparing PS–PED and PAS scores, we find preliminary strong discriminant validity to identify children with FSD (*p* < 0.01). Our results provide evidence on using the 14-item PS–PED as a screening tool for FSD in a clinical sample of children with heterogeneous disease.

## 1. Introduction

Swallowing and feeding disorders (SFD) are described in both toddlers and children with different diagnoses related to developmental disabilities and in those with normal psychomotor development [1,2]. Indeed, the number of these children with feeding and swallowing disorders is estimated to be 25–45%, and the percentage increases to 80% when talking about children with developmental disabilities [3].

As medical and surgical techniques and neonatal therapy have improved, the survival of high-risk newborns has increased significantly, leading to a rise in clinically complex babies. Therefore, the number of children with SFD increases as clinically complex babies increase in number and their hospital stays grow in length [3]. These disorders result from the interaction between different factors and the child’s health, neurological condition, anatomical condition and functional and behavioral development.

The burden of pediatric SFD can be overwhelming for children and their families. In most circumstances, dysphagia foresees more severe illness and worse health outcomes, leading to a longer healthcare need: children with SFD undergo more prolonged hospital stays and more frequent re-entries to hospital, causing a financial strain on the healthcare system and increased use of human resources. On the other hand, proper management of dysphagia reduces the risk of complications and their related costs [3,4,5,6,7]. Early identification of the signs and symptoms of dysphagia is the key to preventing serious complications and comorbidity such as malnutrition, dehydration, respiratory issues and their impact on the child’s and family’s quality of life.

State guidelines for dysphagia practice currently recommend the use of screening as the first step in identifying the risk of swallowing disorders. The screening must be well constructed, have solid psychometric properties and be completed by healthcare professionals in order to identify high-risk individuals and earlier proceed to more thorough assessments [8,9]. Early identification of the child with dysphagia results in decreased comorbidities, improved health status and an early rehabilitation program.

So far, there are a few screening tools for SFD in children built on the idea of adult screenings that involve the patient themselves by submitting them to swallowing tests; however, this is not always possible in children due to pathology, behavior, ability [10], and because it might jeopardize their health status. Some require advanced expertise [10] and are not addressed to all pediatric age groups. As the timing of a swallowing assessment is crucial [11], we aimed to develop a screening tool that can identify early on and thus speed up the assessment of children with SFD admitted to hospital wards. Furthermore, we aimed to create a tool that does not require performing a swallowing test on children and/or food administration, in order to prevent potential complications due to aspiration.

## 2. Methods

The research study was conducted according to the Declaration of Helsinki and good clinical practice. The Bambino Gesù Children’s Hospital Ethics Committee—Rome, Italy, approved the study (protocol number 2352_OPBG_2021). Parents or caregivers of the children were informed about the study objectives and procedures and gave prior written consent.

### 2.1. Study Design

**Figure 1:**
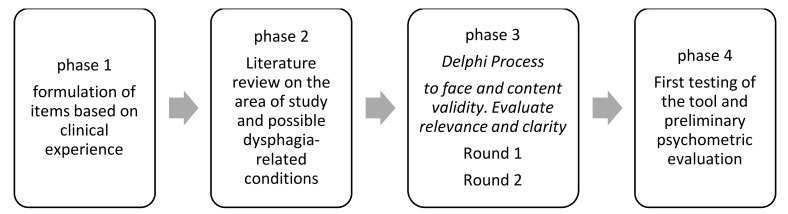
Flow diagram of the development of the components of a PS–PED screening tool.

The development of the Pediatric Screening–Priority Evaluation Dysphagia (PS–PED) followed a step-by-step approach (Figure 1). The first step was to identify the items of the tool from clinical experience. Then, in the second phase, the scientific literature was reviewed to identify evidence on factors associated with the risk of dysphagia/swallowing disorders. Once specific items were identified, according to the collaboration of a panel of experts, a two-round Delphi method (third step) was used to reach a consensus on which items should be inserted in the PS–PED. After that, a pilot study was conducted to test preliminary psychometric properties.

### 2.2. Step 1 and 2: Tool Development and Literature Review

A group formed by researchers and allied healthcare professionals with great experience in SFD first identified a group of possible items to be inserted in the PS–PED. Items were classified into three domains: 1. Clinical history, 2. health status, and 3. feeding condition. Then, a comprehensive review of available literature from the last 10 years was carried out to provide evidence of the correlation between pediatric dysphagia and different health conditions. Search strategies are summarized in Table 1.

Studies were selected by two independent operators (A.C. and G.B.). Any discrepancies or questions on the research papers were settled through discussion or with the assistance of the senior researcher. This process allowed us to identify the items of the screening tool. In this phase, the research group also discussed the scoring process identifying a dichotomous option (Yes = 1–No = 0) as a possible answer. Finally, the structure of the PS–PED was designed, and it was then presented to the panel of experts.

### 2.3. Step 3: Modified Delphi Study

Based on Step 1 and 2, the Delphi method collected expert-based judgments. The Delphi is a planned consensus process that employs a panel of experts to investigate a complex problem by using a sequence of structured statements. The Delphi study was conducted from September 2021 to December 2021. The study employed a two-round Delphi study conducted via email. Participants were informed that they had 15 days to complete the first survey of 19 questions, in which they were asked to assess the clarity of the screening tool in its structure and content. The experts were also assured of anonymity to guarantee the honesty of the answers. Once the first round of the Delphi study was completed, we proceeded with a qualitative and quantitative analysis of the answers provided, to modify and improve the screening tool.

The second survey only consisted of a few questions about the adjustments made to the screening tool and was sent to all the experts but was only filled in by those who had completed the first round of the study. The process ended when the analysis of the answers revealed a common agreement among the participants in the study: consensus on the relevance and clarity of the tool was defined as 80% agreement [12].

To analyze and catalog the data, answers for the closed-ended questions were collected in an Excel matrix, whereas the open-ended questions data were coded for redundancy.

### 2.4. Panel Information

The panel members were selected according to expertise and competence criteria. They were all Italian healthcare professionals with a thorough knowledge of this topic and at least five years of clinical experience in FSD. The interdisciplinary expert panel of 50 professionals included 20 speech–language pathologists, 10 speech–language pathologists with a Master’s degree in feeding and swallowing disorders, 10 nurses, 5 medical doctors and 5 physiotherapists with a Master’s degree in respiratory therapy. The average experience with FSD of the Delphi participants was 7.3 years.

This Delphi aimed to reach a common consensus on the items’ thoroughness and clarity.

### 2.5. Step 4: Pilot Study

Once the screening tool was defined, we conducted a pilot study to test it. The screening tool was administered to 60 children between 13 months and 16 years old staying at the Bambino Gesù Pediatric Hospital in Palidoro, Rome; these children (25 boys and 35 girls) had different underlying pathologies. The study included all the children who had undergone or were about to undergo an instrumental swallowing assessment via the videofluoroscopy swallow study (VFSS).

All the necessary information for the PS–PED compilation was retrieved from each child’s medical record. Once the information was collected, the answers were summed together, and a total score was established for each child. Frequency tables, mean and standard deviation scores were used as descriptive statistics.

Cronbach’s alpha was used to evaluate the internal consistency of the PS–PED. As Nunally [13] reported, a satisfactory index of a scale’s homogeneity should have an alpha coefficient > 0.70.

The Penetration Aspiration Scale (PAS) was used to classify the results of the VFSS and to measure construct validity. This scale has been used since 1996 in the assessment of dysphagia; it has become the gold standard method used by clinicians and researchers to describe and measure the depth and response to airway invasion during videofluoroscopy [4]. It was developed by Rosembek et al. and has 8 levels indicating the severity of the bolus airway invasion during the VFSS; it captures where the bolus is placed after the swallowing act to objectify the information observed. However, as this work aimed to identify children at risk for dysphagia, we divided the results from the PAS into two groups A and B. All VFSS that resulted in a PAS level 1 (material does not enter the airways) were included in group A; all other PAS levels from 2 to 8 were included in group B, as these correspond to penetration/aspiration of material into the airway. The scoring was carried out by an experienced radiologist, a speech pathologist, and an expert in the field of deglutology, who did not administer the screening tool. Pearson’s correlation coefficient was used to investigate the relationship between PS–PED and PAS. Pearson’s correlation coefficients range from 0 (indicating no relationship between variables) to 1 (indicating a perfect relationship), and values were interpreted as follows: 0.3 indicated a weak relationship, 0.3–0.69 indicated a moderate relationship, and values >0.7 indicated a strong relationship [14].

Finally, an independent sample *t*-test was used to verify preliminary discriminant validity and compare the mean of PS–PED according to PAS analysis. Significance was set at <0.05 95% CI.

### 2.6. Translation and Cultural Adaptation

This screening was developed in Italian. However, we also provide a translation and adaptation into English to reach an international audience. Seven experts were involved. First, two bilingual people (Italian and English) translated the Italian version into two English versions (EV1 and EV2). Both versions were then compared by a panel of experts that agreed on the first English version of the PS–PED.

During step 2, two other experts (IV1, IV2) made a back translation of the English version and compared it to the final Italian version (IVF). The final English version was produced in the third and last step, which we report on in this study.

## 3. Results

### 3.1. Literature Review

**Figure 2:**
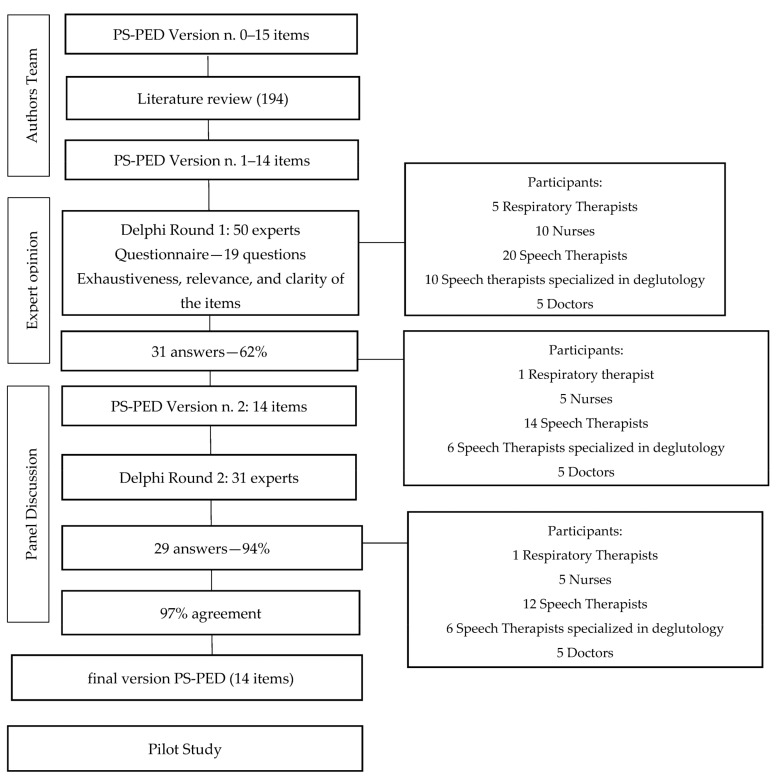
Reports each step for the development of PS–PED.

After removing duplicates, the scoping review search retrieved 1624 articles from electronic databases. From the first screening, a total of 194 articles were selected based on title and abstract. During the second step, the full articles were read to establish their relevance and pertinence concerning dysphagia/swallowing disorders in children. The comprehensive literature review confirmed some items as significant for identifying FSD in children and overruled others. Fourteen items were positively identified and classified into three domains: 1. Clinical history, 2., health status and 3., feeding condition. We did not include the item on the presence or absence of drooling as the articles did not show whether it was related to dysphagia in a dichotomous way. No evidence was found that could justify a relationship between the use of a suction machine/aspirator with swallowing disorders/dysphagia; however, considering their experience the authors decided to include it in the screening and have it evaluated by experts. Finally, from the literature study concerning parenteral/enteral nutrition and consistency and unsuitable food, the authors concluded that using parenteral or enteral nutrition leads to unacceptance of textures and unsuitable food (Figure 2).

### 3.2. Modified Delphi study

A first questionnaire was sent to 50 professionals who formed the panel of experts, but only 31 (62%) questionnaires were received. A qualitative and quantitative analysis of the answers was also carried out, allowing us to modify and improve the screening tool. This analysis showed that negative items could be difficult to understand, could be confusing or make the tool’s administration less efficient, so we reworded some items to be more straightforward and clearer.

There was a clear focus on the actual phrasing of the items, while both the scoring and the tool’s structure were clear to most of the experts. Moreover, it was considered significant to view the answers according to the experts’ professional role; nearly all experts, regardless of their professional role, strongly agreed and believed that no important items that could make the tool more effective had been omitted.

Second round: the second questionnaire was sent to the panel experts who had completed the first round of the study. Twenty-eight participants completed the second questionnaire resulting in a response rate of 94%. All the answers of the second round were analyzed by focusing on areas of agreement. The new version of the tool was found simpler and faster to administer by 97% of the panel experts. The screening tool was modified according to the feedback scores and comments from the panel of experts who were asked to assess the form and quality validity. The final version of the tool received very high average scores, highlighting a strong opinion of the instrument. The final version of the PS–PED is described in Table 2.

### 3.3. Pilot Study

A preliminary statistical analysis was made to test the new screening tool on the population of interest. 60 children, 24 boys and 36 girls, admitted to the Bambino Gesù Pediatric Hospital were assessed with the screening tool. They had different diagnoses, listed in the table according to an internationally recognized classification [15,16], and numbered from 1 to 5 to simplify statistics. Sample characteristics are reported in Table 3.

### 3.4. Data Analysis

At the outset of our investigation, we reported the frequency and percentage of presence (yes) and absence (no) for each variable reported in the PS–PED, reported in Table 4.

Internal consistency estimates revealed Cronbach’s alpha coefficients of 0.731. As reported in Table 5, item–total analysis showed that all items positively contribute to determining the scale’s total score.

**Figure 3:**
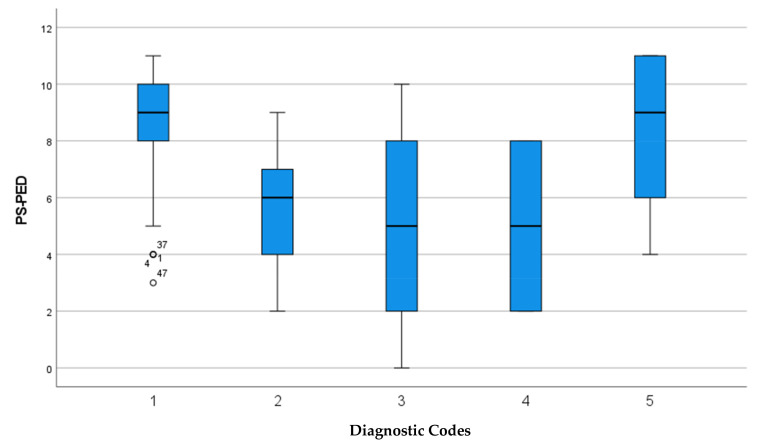
Differences in PS-PED score across different diagnostic groups. 1—Neurological and neuromuscular conditions; 2—Structural anomalies of the digestive and respiratory systems; 3—Others, 4—Cardiac disorders; 5—Genetic syndromes.

We also investigated differences in the scoring of PS–PED across different diagnoses. Groups 1 (neurological and neuromuscular conditions) and 5 (genetic syndromes) showed a higher score of PS–PED than other groups. Figure 3 synthetizes the results of PS–PED for each diagnostic group.

**Figure 4:**
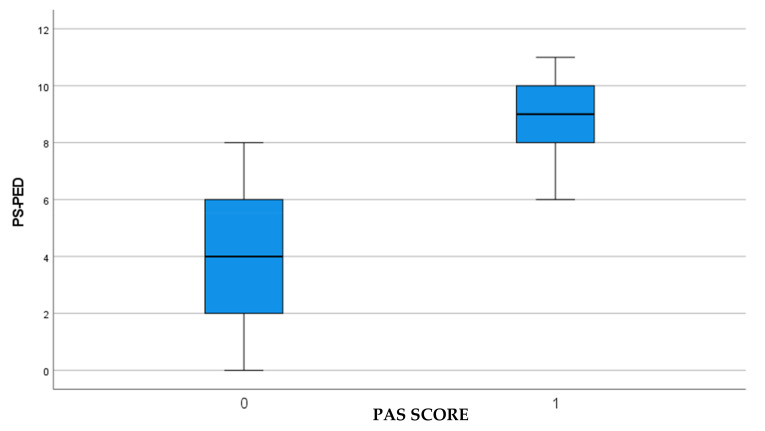
PS-PED score distribution according to PAS score: 0—Negative score for swallowing disorders; 1—Positive score for swallowing disorders (penetration/aspiration).

As for construct validity estimates, PS–PED showed a positive correlation with the PAS score with a Pearson correlation coefficient of 0.824 (*p* < 0.01). To verify the preliminary discriminant validity of the PS–PED we divided the PAS score into a dichotomic group (0 = negative PAS; 1 = positive PAS) and found significant differences in PS–PED. Results are summarized in Table 6 and Figure 4.

## 4. Discussion

This study aimed to develop a screening for assessing dysphagia in children. The research group conducted a rigorous method; the Dysphagia Team’s clinical experience led to the development of the items, which were later studied and analyzed through a scientific literature review and a two-round Delphi survey, which achieved 97% consensus. This confirmed the selected items’ relevance and the instrument’s thoroughness.

The ability to swallow is a basic human function, and causes of dysfunction can be various and multi-factorial. The focus on these issues has increased over the past few years.

J. Horton et al., in a 2018 article, highlighted how the rate of hospitalized children with medical conditions that lead to dysphagia or consequences of dysphagia itself had increased dramatically and will increase further over time. This is due to improvements in the early care and treatment of children with significant comorbidities, such as cerebral palsy, chronic respiratory diseases, congenital malformations, etc., that carry a congenitally or iatrogenically impaired swallowing function [3].

The prevalence of dysphagia will rise in pediatric hospitals precisely because of the type of patients being admitted. The same authors point out that pediatric dysphagia requires multidisciplinary care coordinated by a specialized team, which will require significant use of human resources [3] and healthcare expenses [4].

As J. Horton’s study suggests, this has been our experience within the Bambino Gesù Pediatric Hospital. Over the last 10 years, there has been a higher number of clinically complex children’s admission, dramatically increasing the demand for dysphagia consults and the related instrumental exams requested. This has resulted in the rise of resources needed and healthcare costs.

Among other things, these data led us to develop a screening tool for the early identification of signs and symptoms of dysphagia in children: we aim to make the care of infants and children with dysphagia and eating disorders more efficient and effective.

Providing early intervention to reduce the negative outcomes of FSD is well documented in the literature [11,17,18,19]. According to the European Society for Swallowing Disorders [20] and the American Speech–Language–Hearing Association (ASHA 2016), screening tools must be the first step when treating patients with dysphagia. They must be able to identify those at risk with high sensitivity and specificity, be rapid and cheap, and present a low risk for the patient [11].

Ebru Umay et al., in a 2022 review of best practice recommendations for pediatric dysphagia, highlighted three formal screening tests (Neo—EAT, Pedi EAT and Pediatric Dysphagia risk screening test), all of which are age-specific [18]. So far, in the literature, there are limited screening tools for feeding and swallowing disorders, which mainly involve direct clinical assessments of the patient or are based on adult screening tools such as the 3-ounce (90-cc) water swallow challenge [21].

Our study aimed to develop a screening tool that meets all the required criteria and includes all age groups and diagnoses of children admitted to a Pediatric Hospital. The PS–PED is designed according to the International Scientific Community’s criteria for screening for swallowing disorders. To be appropriate, a screening tool that identifies swallowing disorders should be rapid, minimally invasive and should be able to determine the likelihood of dysphagia and to establish whether a further swallowing assessment is needed or whether oral feeding is safe for the patient [22].

The PS–PED can be administered in less than 10 min and does not require any specific education in swallowing disorders, since the predictive markers for dysphagia are related to the patient’s clinical and feeding history and not to the dysphagia itself. It does not involve administering any food to the patient.

Preliminary statistics show good internal consistency and construct validity. PS–PED also showed good discriminant validity for the identification of dysphagia. Additionally, the PS–PED makes no distinction between age and pathology and can be used by physicians when taking a medical history and by healthcare professionals who have access to the patient’s medical history. All the items have a “yes” or “no” answer and were designed to limit the subjective nature of the answers, for example, the presence or absence of tracheostomy, or alternative feeding.

The Consensus-based Standards for selecting health status Measurement Instruments (COSMIN) define the diagnostic strength of a screening tool by comparing the tool with the gold-standard exam accepted by the scientific community. This is defined as a criterion of validity [17]. Therefore, we compared the PS–PED results with the VFSS, which is considered the gold standard, enhancing its validity and consensus. The PS–PED showed good preliminary psychometric properties, in terms of internal consistency (Cronbach’s Alpha 0.731) and concurrent validity (Pearson’s correlation coefficient 0.824). Furthermore, PS–PED showed good preliminary discriminant validity for detecting children with SFD.

## 5. Limits

The limits of this study include the small sample of patients who were administered the PS–PED and a preliminary statistical analysis. The screening tool was also administered to patients with different diagnoses; however, it would be interesting to test its validity according to pathology separately. Currently, the tool has only been used by speech–language pathologists working in the Dysphagia Unit. It would be useful to evaluate its reliability when administered by different health professionals.

## 6. Future Prospects

This study is just an initial development of the screening tool, which has yet to be validated. However, our findings from the preliminary statistical analysis are encouraging and urge us to move towards a larger sample of children to validate the tool properly. Therefore, we plan to continue this work by administering the tool to a larger sample of children to obtain more statistically significant data. This would allow us to demonstrate the reliability, validity, discriminative and assessment capacity of the PS–PED. Indeed, the success of the survey is believed to depend on the professional world’s consensus and, above all, on its psychometric quality.

## 7. Conclusions

This study identified a 14-item hospital-based Pediatric Screening tool for FSD, Priority Evaluation Dysphagia (PS–PED). The psychometric properties strongly highlight solid reliability and prove the screening tool’s usability

## Figures and Tables

**Table 1:** Electronic Databases and Search strategy.

Electronic Databases
PubMed, Scopus, Web of Science, and CINAHL
**Search Statements**
Swallowing disorders OR dysphagia AND infants AND children AND tracheostomy; swallowing disorders OR dysphagia AND infants AND children AND congenital heart disease; swallowing disorders OR dysphagia AND infants AND children AND neurologic diagnosis; swallowing disorders OR dysphagia AND infants AND children AND cerebral palsy; swallowing disorders OR dysphagia AND infants AND children AND gastroesophageal reflux; swallowing disorders OR dysphagia AND infants AND children AND feeding tube; swallowing disorders OR dysphagia AND infants AND children AND time mealtime; swallowing disorders OR dysphagia AND infants AND children AND lung infection; swallowing disorders OR dysphagia AND infants AND children AND esophageal atresia; swallowing disorders OR dysphagia AND infants AND children AND epilepsy; swallowing disorders OR dysphagia AND infants AND children AND delay feeding abilities.

**Table 2:** Final English version of the Priority Evaluation Dysphagia (PS–PED).

Domain	Number of Items	Items	Response SCORE	
			YES	NO
Clinical history	4	1. Neurological diagnosis	1	0
		2. Epilepsy medications	1	0
		3. Heart disease	1	0
		4. Structural anomalies of the digestive and respiratory systems	1	0
Health status	7	5. Tracheal tube	1	0
		6. Decreased Alertness	1	0
		7. Malnutrition and/or poor growth	1	0
		8. Recurrent respiratory tract infections	1	0
		9. Use of the suction machine/aspirator	1	0
		10. Lack of head control and/or postural instability	1	0
		11. gastrointestinal diseases (gag reflex, vomit, constipation, GERD)	1	0
Feeding conditions	3	12. Parenteral/Enteral nutrition (nasogastric tube, gastrostomy tube, etc.)	1	0
		13. Feeding with consistency and unsuitable food for the child’s development stage	1	0
		14. Prolonged mealtime (over 50 min)	1	0

**Table 3:** Demographic and clinical characteristics of participants.

Characteristics	n	%
Gender		
Males	24	40%
Females	36	60%
Range age	0.3 to 17 years	
Mean age		
Standard deviation		
Medical Diagnosis		
1. Neurological Neuromuscular	27	45%
2. Structural anomalies digestive and respiratory systems	9	15%
3. Other	6	10%
4. Heart Disease	4	6%
5. Genetic Syndrome	12	20%

**Table 4:** Descriptive analysis of the PS–PED.

Domain	Items	No		Yes	
		N	%	N	%
Clinical History	1. Neurological diagnosis	24	40.7	35	59.3
	2. Epilepsy medications	42	71.2	17	28.8
	3. Heart disease	50	84.7	9	15.3
	4. Structural anomalies of the digestive and respiratory systems	32	54.2	27	45.8
Health status	5. Tracheal tube	44	74.6	15	25.4
	6. Decreased Alertness	51	86.4	8	13.6
	7. Malnutrition and/or poor growth	32	54.2	27	45.8
	8. Recurrent respiratory tract infections	19	32.2	40	67.8
	9. Use of the suction machine/aspirator	26	44.1	33	55.9
	10. Postural instability and lack of control	24	40.7	35	59.3
	11. gastrointestinal diseases (gag reflex, vomit, constipation, GERD)	18	30.5	41	69.5
Feeding conditions	12. Parenteral/Enteral nutrition (nasogastric tube, gastrostomy tube, etc.)	18	30.5	41	69.5
	13. feeding with consistency and unsuitable food for the child’s development stage	6	10.2	53	89.8
	14. Prolonged mealtime (over 50 min)	8	13.6	51	86.4

**Table 5:** Internal consistency of the PS–PED.

	Mean	Std. Deviation	Scale Mean if Item Deleted	Scale Variance if Item Deleted	Corrected Item-Total Correlation	Cronbach’s Alpha if Item Deleted
Item 1	0.59	0.495	6.73	7.270	0.316	0.708
Item 2	0.29	0.457	7.03	7.551	0.239	0.717
Item 3	0.15	0.363	7.17	8.350	−0.058	0.742
Item 4	0.46	0.502	6.86	8.671	−0.190	0.769
Item 5	0.25	0.439	7.07	8.133	0.014	0.741
Item 6	0.14	0.345	7.19	7.637	0.316	0.708
Item 7	0.46	0.502	6.86	7.257	0.314	0.708
Item 8	0.68	0.471	6.64	6.440	0.710	0.656
Item 9	0.56	0.501	6.76	6.701	0.543	0.677
Item 10	0.59	0.495	6.73	6.787	0.514	0.681
Item 11	0.69	0.464	6.63	6.928	0.498	0.685
Item 12	0.69	0.464	6.63	6.893	0.513	0.683
Item 13	0.90	0.305	6.42	7.317	0.576	0.688
Item 14	0.86	0.345	6.46	7.149	0.591	0.682
Total	7.36	2.92				

**Table 6:** Differences between PS–PED mean score and PAS.

		N	Mean	Std. Deviation	Std. Error Mean	*t*-Test
PS–PED	PAS Negative	21	4.14	2.081	0.454	<0.01
	PAS Positive	38	9.13	1.398	0.227	

## Data Availability

The data presented in this study are available on request from the corresponding author.

## References

[1] Duffy K.L. (2018). Dysphagia in Children. Curr. Probl. Pediatr. Adolesc. Health Care.

[2] Lefton-Greif M., Arvedson J. (2016). Pediatric Feeding/Swallowing: Yesterday, Today, and Tomorrow. Seminars in Speech and Language.

[3] Horton J., Atwood C., Gnagi S., Teufel R., Clemmens C. (2018). Temporal Trends of Pediatric Dysphagia in Hospitalized Patients. Dysphagia.

[4] Steele C.M., Mukherjee R., Kortelainen J.M., Pölönen H., Jedwab M., Brady S.L., Theimer K.B., Langmore S., Riquelme L.F., Swigert N.B. (2019). Development of a Non-Invasive Device for Swallow Screening in Patients at Risk of Oropharyngeal Dysphagia: Results from a Prospective Exploratory Study. Dysphagia.

[5] Andrade P.A., Santos C.A.d., Firmino H.H., Rosa C.d.O.B. (2018). The Importance of Dysphagia Screening and Nutritional Assessment in Hospitalized Patients. Einstein.

[6] Perry L., Love C.P. (2001). Screening for Dysphagia and Aspiration in Acute Stroke: A Systematic Review. Dysphagia.

[7] Speyer R., Cordier R., Parsons L., Denman D., Kim J.-H. (2018). Psychometric Characteristics of Non-Instrumental Swallowing and Feeding Assessments in Pediatrics: A Systematic Review Using COSMIN. Dysphagia.

[8] American Speech-Language-Hearing Association (ASHA) 2019. https://www.asha.org/.

[9] European Society for Swallowing Disorders 2012. https://essd.org/.

[10] Etges C.L., Barbosa L.D.R., de Almeida M.C., Cardoso F. (2020). Development of the Pediatric Dysphagia Risk Screening Instrument (PDRSI). Codas.

[11] Brodsky M.B., Mayfield E.B., Gross R.D. (2019). Clinical Decision Making in the ICU: Dysphagia Screening, Assessment, and Treatment. Seminars in Speech and Language.

[12] Colombo C., Catastini P., Brivio A., Acone B., Dang P., Quattrucci S. (2018). Delphi Poll to Assess Consensus on Issues Influencing Long-Term Adherence to Treatments in Cystic Fibrosis among Italian Health Care Professionals. Patient Prefer. Adherence.

[13] Hundleby J.D., Nunnally J. (2006). Psychometric Theory. Am. Educ. Res. J..

[14] Monticone M., Galeoto G., Berardi A., Tofani M. (2021). Psychometric Properties of Assessment Tools. Measuring Spinal Cord Injury.

[15] Arvedson J.C. (2008). Assessment of Pediatric Dysphagia and Feeding Disorders: Clinical and Instrumental Approaches. Dev. Disabil. Res. Rev..

[16] Hartnick C.J., Cotton R.T. (2000). Congenital Laryngeal Anomalies. Otolaryngol. Clin. N. Am..

[17] Onesimo R., Sforza E., Giorgio V., Rigante D., Kuczynska E., Leoni C., Agazzi C., Limongelli D., Cerchiari A., Tartaglia M. (2023). Predicting the clinical trajectory of feeding and swallowing abilities in CHARGE syndrome. Eur. J. Pediatr..

[18] Umay E., Eyigor S., Giray E., Karadag Saygi E., Karadag B., Durmus Kocaaslan N., Yuksel D., Demir A.M., Tutar E., Tikiz C. (2022). Pediatric Dysphagia Overview: Best Practice Recommendation Study by Multidisciplinary Experts. World J. Pediatr..

[19] Banzato A., Cerchiari A., Pezzola S., Ranucci M., Scarfò E., Berardi A., Tofani M., Galeoto G. (2022). Evaluation of the Effectiveness of Functional Chewing Training Compared with Standard Treatment in a Population of Children with Cerebral Palsy: A Systematic Review of Randomized Controlled Trials. Children.

[20] Speyer R., Cordier R., Farneti D., Nascimento W., Pilz W., Verin E., Walshe M., Woisard V. (2022). White Paper by the European Society for Swallowing Disorders: Screening and Non-Instrumental Assessment for Dysphagia in Adults. Dysphagia.

[21] Suiter D.M., Leder S.B., Karas D.E. (2009). The 3-Ounce (90-Cc) Water Swallow Challenge: A Screening Test for Children with Suspected Oropharyngeal Dysphagia. Otolaryngol.-Head Neck Surg..

[22] Jiang N., Sung C.K., Damrose E.J. (2017). Improvement in the Reflux Symptom Index Following Surgery for Cricopharyngeal Dysfunction. J. Voice.

